# Supplementary material for: Splice-Junction-Based Mapping of Alternative Isoforms in the Human Proteome
Source: Cell Rep. Author manuscript; Available in PMC 2020 Jan 15. (PMC6961840; doi:10.1016/j.celrep.2019.11.026)
Supplement: 3 [file NIHMS1546469-supplement-3.zip › DF2/PXD009737/TestisLysC-115-Q9ULH0-TSLPRDQNNGLGSGPAPGPVVLLNSLNVDAVCEK.pdf]

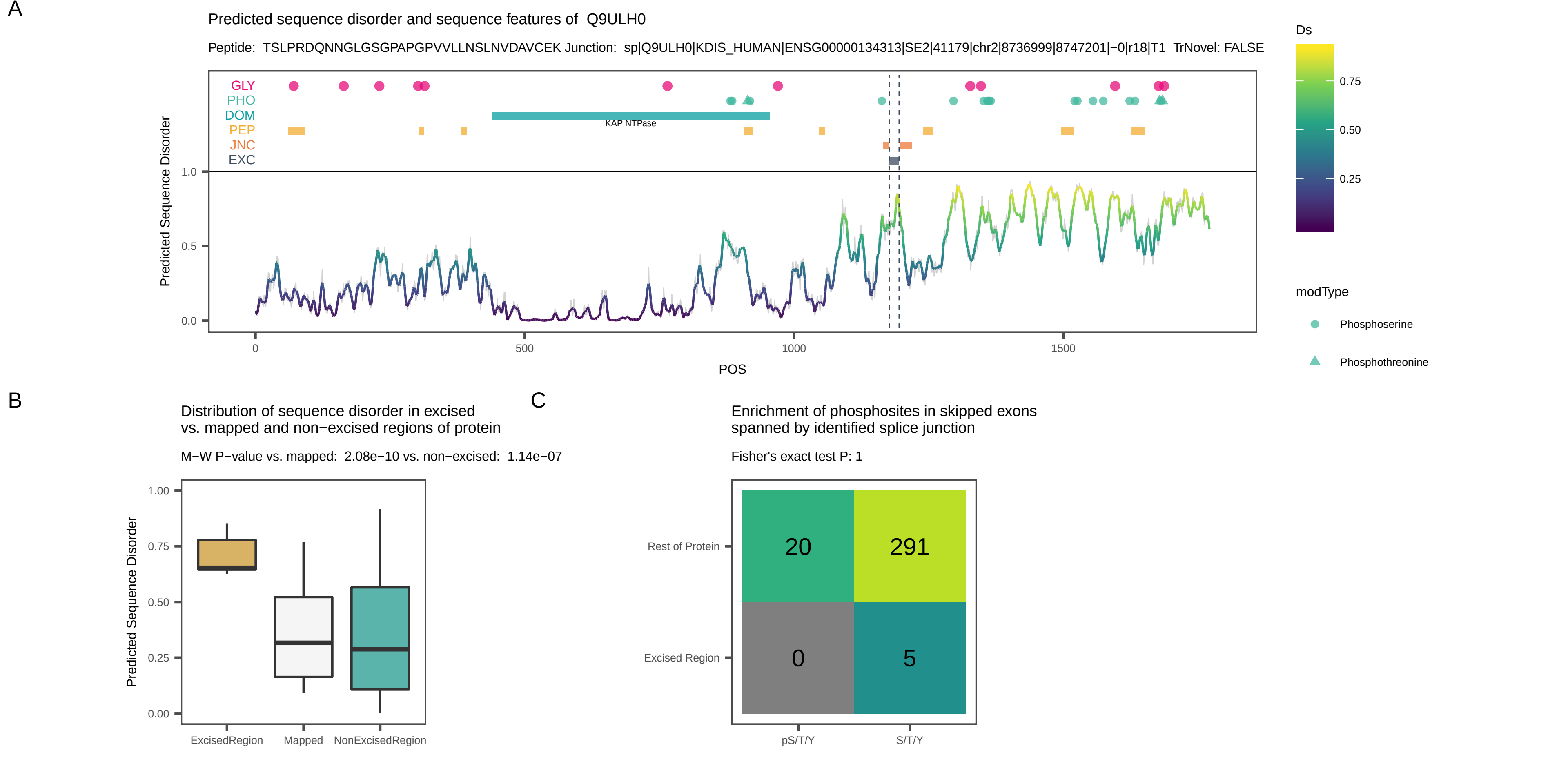
A
Predicted sequence disorder and sequence features of Q9ULH0
Peptide: TSLPRDQNNGLGSGPAPGPVVLLNSLNVDAVCEK Junction: sp|Q9ULH0|KDIS_HUMAN|ENSG00000134313|SE2|41179|chr2|8736999|8747201|−0|r18|T1 TrNovel: FALSE
GLY
PHO
DOM
PEP
JNC
EXC
KAP NTPase
Predicted Sequence Disorder
1.0
0.5
0.0
0
500
1000
1500
POS
Ds
0.75
0.50
0.25
modType
Phosphoserine
Phosphothreonine
B
Distribution of sequence disorder in excised vs. mapped and non−excised regions of protein
M−W P−value vs. mapped: 2.08e−10 vs. non−excised: 1.14e−07
Predicted Sequence Disorder
1.00
0.75
0.50
0.25
0.00
ExcisedRegion
Mapped
NonExcisedRegion
C
Enrichment of phosphosites in skipped exons spanned by identified splice junction
Fisher's exact test P: 1
Rest of Protein
Excised Region
20
291
0
5
pS/T/Y
S/T/Y
